# A Randomized Comparison of Delivered Energy in Cardioversion of Atrial Fibrillation: Biphasic Truncated Exponential Versus Pulsed Biphasic Waveforms

**DOI:** 10.3390/diagnostics11061107

**Published:** 2021-06-17

**Authors:** Elina Trendafilova, Elena Dimitrova, Jean-Philippe Didon, Vessela Krasteva

**Affiliations:** 1 Intensive Cardiology Care Unit, Cardiology Clinic, National Cardiology Hospital, 65 Konyovitza Str., 1309 Sofia, Bulgaria; elitrendafilova@abv.bg (E.T.); elena.sv@gmail.com (E.D.); 2 Schiller Médical SAS, 4 rue L. Pasteur, F-67160 Wissembourg, France; jean-philippe.didon@schiller.fr; 3 Institute of Biophysics and Biomedical Engineering, Bulgarian Academy of Sciences, Acad. G. Bonchev Str. Bl 105, 1113 Sofia, Bulgaria

**Keywords:** biphasic waveform defibrillation, atrial fibrillation, cardioversion, delivered energy, efficacy, safety, high-sensitivity troponin

## Abstract

A few randomized trials have compared impedance-compensated biphasic defibrillators in clinical use. We aim to compare pulsed biphasic (PB) and biphasic truncated exponential (BTE) waveforms in a non-inferiority cardioversion (CVS) study. This was a prospective monocentric randomized clinical trial. Eligible patients admitted for elective CVS of atrial fibrillation (AF) between February 2019 and March 2020 were alternately randomized to treatment with either a PB defibrillator (DEFIGARD TOUCH7, Schiller Médical, Wissembourg, France) or a BTE high-energy (BTE-HE) defibrillator (LIFEPAK15, Physio-Control Inc., Redmond, WA, USA). Fixed-energy protocol (200–200–200 J) was administered. CVS success was accepted if sinus rhythm was restored at 1 min post-shock. The study design considered non-inferiority testing of the primary outcome: cumulative delivered energy (CDE). Seventy-three out of 78 randomized patients received allocated intervention: 38 BTE-HE (52%), 35 PB (48%). Baseline characteristics were well-balanced between groups (*p* > 0.05). Both waveforms had similar CDE (mean ± standard deviation, 95% confidence interval): BTE-HE (253.9 ± 120.2 J, 214–293 J) vs. PB (226.0 ± 109.8 J, 188–264 J), *p* = 0.31. Indeed, effective PB shocks delivered significantly lower energies by mean of 25.6 J (95% CI 24–27.1 J, *p* < 0.001). Success rates were similar (BTE-HE vs. PB): 1 min first-shock (84.2% vs. 82.9%), 1 min CVS (97.4% vs. 94.3%), 2 h CVS (94.7% vs. 94.3%), 24 h CVS (92.1% vs. 94.3%), *p* > 0.05. Safety analysis did not find CVS hazards, reporting insignificant changes of myocardial-specific biomarkers, transient and rare ST-segment deviations, and no case of harmful tachyarrhythmias and apnea. Cardioversion of AF with fixed-energy protocol 200–200–200 J was highly efficient and safe for both PB and BTE-HE waveforms. These similar performances were achieved despite differences in the waveforms’ technical design, associated with significantly lower delivered energy for the effective PB shocks. Clinical Trial Registration: Registration number: NCT04032678, trial register: ClinicalTrials.gov.

## 1. Introduction

External electrical cardioversion (CVS) is a standard treatment for ventricular and supraventricular arrhythmias [1,2]. Different biphasic waveforms have been shown to be more effective and safer at lower energies than monophasic ones [3,4,5,6,7].

A few clinical CVS trials have compared the efficacy and safety of biphasic defibrillators using industry standard waveforms, namely rectilinear biphasic (RB), biphasic truncated exponential (BTE) or pulsed biphasic (PB) [8,9,10,11,12,13,14,15,16,17]. Impedance compensation of all defibrillation waveforms is required by standards, which tolerate a deviation of ±15% in delivered energy [18]. The same RB, BTE and PB energy settings are, however, supplied by different currents, voltages and timing control [19,20,21]. This might have an effect on the outcome [21,22,23,24]. Therefore, the efficacy analysis is important to consider the real-life pulse characteristics and energy deviation which can be accurately measured [19,20,25,26]. At the same time, the efficacy analysis should take into account different definitions of CVS success, which varies from post-shock restoration of sinus rhythm at 5 s [9,11], 30 s [15] to 4 h [12], and most often 1 min [8,10,13,14].

The safety analysis in clinical CVS trials analyzes the harmful effects of electric shocks by assessment for pain and erythema, measurement of high-sensitivity troponin I (hsTnI), creatine phosphokinase (CPK), creatine kinase isoenzyme MB (CK-MB), monitoring for post-shock arrhythmias (tachyarrhythmia and bradyarrhythmia), and early and late ST-segment deviation [6,27,28,29]. The safety profile of electric shocks can be better assessed in CVS for atrial fibrillation (AF) than in cardiac arrest because the myocardium is perfused [30,31].

Scarce clinical trials have been found to study the efficacy and safety of PB waveform in CVS [12,13,17]. A systematic literature review on CVS efficacy [32] demonstrates that the dose–response curves of PB, BTE with high energy (BTE-HE) [10,11,12] and BTE with low energy (BTE-LE) [8,15] concentrate within 85–95% success rate and 300–500 J cumulative energy, despite major disparities found between published studies (success definition, rhythm inhomogeneity, different population sizes, different selected energy levels, etc.). The validity of significantly deviating efficacy of PB waveform in one of the surveyed studies is questionable [12], linked to the use of a defective device. Indeed, our recent CVS trial demonstrates no superiority of BTE-LE over PB waveform with an excellent safety profile without post-shock myocardial injuries [13]. More data from randomized studies, assessing the efficacy and safety of PB waveform, are required according to international guidelines [33].

This study aims for the first time to test the non-inferiority of cumulative delivered energy of PB vs. BTE-HE waveforms in a prospective randomized clinical CVS trial of AF patients. Additional clinical implications are demonstrated by statistical test results on numerous secondary efficacy and safety outcomes.

## 2. Methods

### 2.1. Study Design and Setting

This was a prospective randomized single-blinded trial with parallel assignment (alternating design) of AF patients to elective CVS with either PB or BTE-HE defibrillation waveforms. Following the order of patient admittance in the Intensive Coronary Care Unit (ICCU), Clinic of Cardiology, National Cardiology Hospital (NCH), Sofia, Bulgaria, the attending cardiologist assigned the odd and even eligible patients to the defibrillators, embedding the waveforms in arm 1 (PB) and arm 2 (BTE-HE), respectively. Patients were blinded to the intervention; while cardiologists were not blinded to the used defibrillator, however, they could not control the order of patient admittance in ICCU-NCH. The alternate randomization design was applied to equalize the number of subjects on each treatment. This study was not designed to control for sex, age, comorbidity, type of device used for CVS, cumulative energy delivered during shocks, or the number of shocks administered. Randomization was performed to limit these and any other confounding factors.

### 2.2. Intervention

CVS interventions with PB waveform were performed with the automated external defibrillator DEFIGARD Touch 7 (DGT7, Schiller Médical, Wissembourg, France). Alternatively, BTE-HE waveform was delivered using the defibrillator LIFEPAK 15 (LP15, Physio-Control Inc., Redmond, WA, USA). Both commercial defibrillators used in R-synchronization mode were approved for clinical use. The self-adhesive pads were identically placed for both devices in the anterior-lateral position according to respective manufacturers’ instructions for use.

**Figure 1:**
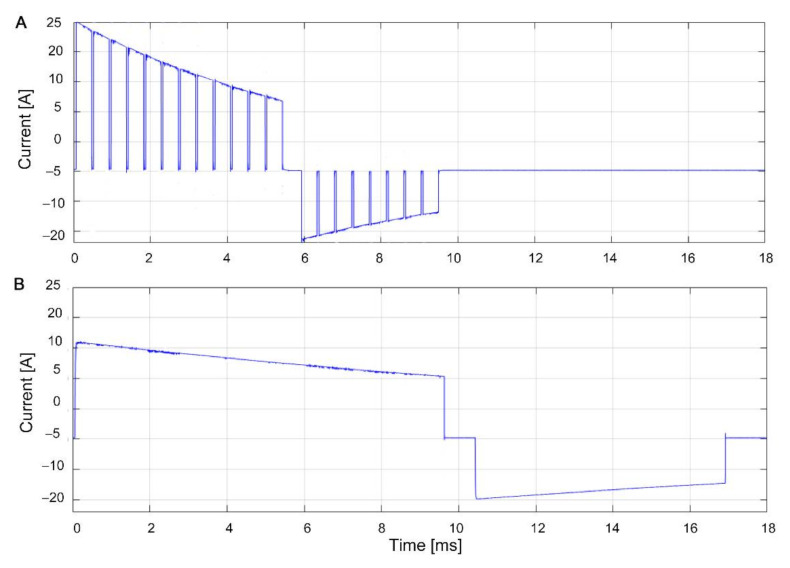
Recorded currents of studied defibrillation waveforms: (**A**) (PB generated with DGT7), (**B**) (BTE-HE generated with LP15). Both waveforms were digitized with the Defimpulse recorder under the same test conditions: selected energy (200 J) and patient impedance (100 Ω).

In both study arms, defibrillation waveforms were digitized in real-time during the shock by a dedicated measurement device (Defimpulse recorder) [25]. It recorded high-resolution currents and voltages in all phases of the defibrillation pulse with sampling rate of 5 MHz, and resolution of 2 V (voltages) and 0.049 A (currents). The energy of each shock was computed using voltage and current samples measured during the shock. The Defimpulse recorder also measured the transthoracic impedance between the defi-pads as a baseline quantity 10 s before each CVS intervention. This device provided independent data of the delivered shock waveform and transthoracic impedance, whatever the studied defibrillator, its waveform technology, impedance compensation or electrodes used. Such data are typically internally stored in defibrillators and not accessible for external evaluation of the delivered shock characteristics. Diagrams of the recorded real pulses for both PB and BTE-HE waveforms are shown in Figure 1. The Defimpulse recorder was an autonomous device and expert assistance was not needed during CVS interventions. The use of the Defimpulse recorder in ICCU-NCH was approved by the NCH Ethical Committee (project identification code, date: № 01, 03 June 2008) for clinical use.

### 2.3. Study Population and Ethics

All adult patients admitted for elective CVS of AF in ICCU-NCH were eligible for the study following hospital standard admission procedures. Informed consent was obtained from all patients before CVS. The study was conducted in accordance with the principles of the Declaration of Helsinki. All patients received the standard hospital procedures during CVS, accepted in the NCH and approved by the NCH Ethical Committee (project identification code, date: № 2902-2536, 23 July 2018). Data anonymization policy was respected. In order to ensure medical confidentiality, no information about the patient (intervention place and date, age, gender, name, diagnosis, etc.) was entered in the defibrillators. An identification number was given to each patient. This number and all study data were proprietary to the principal investigator.

Patients were included in the study if they were aged ≥18 years and had symptomatic (EHRA score 2–4) persistent AF or symptomatic first detected AF or persistent AF after successful causal therapy. Patients were excluded if they did not meet the inclusion criteria, had atrial flutter or were unable/unwilling to sign the informed consent.

### 2.4. Cardioversion Protocol

In both study arms, patients were treated identically with a maximum of 3 shocks at the fixed-energy setting (200–200–200 J). The protocol was stopped at successful CVS (sinus rhythm at 1 min post-shock), otherwise after the third shock. The use of repeated equal energy shocks is justified by the probabilistic nature of defibrillation and is likely to minimize the number of high energy shocks.

CVS interventions were performed in ICCU-NCH by a cardiologist and anesthesiologist. The anesthesia was provided with slow intravenous injection of Propofol, adjusted individually to reach deep sedation (Cook’s scale points <7). Atropine was indicated at the discretion of the attending physician: 0.5 mg Atropine sc 15–30 min before CV, and Atropine qs iv after CV in case of severe or symptomatic bradycardia. Appropriate standard anticoagulation with unfractionated heparin or acenocoumarol or direct oral anticoagulants were applied before and after CVS.

### 2.5. Baseline and Follow-Up Measurements

Baseline variables were collected before randomization during the pre-cardioversion enrollment. All patients underwent TEE within 1–5 days before CVS. Routine blood tests and biomarkers (CK-MB, hsTnI) were measured within 24 h before CVS. Demographic and pre-treatment data (concomitant diseases, medications during 7 days before CVS, left atrium and left ventricle dimensions and volumes, ejection fraction by TEE, AF duration, etc.) were collected at baseline.

Standard 12-lead electrocardiogram (ECG), blood pressure (BP) and heart rate (HR) were measured just before and 3 times after CVS (1–5 min, 1 h, 2 h). Peripheral ECG was continuously recorded during CVS. Follow-up period in ICCU was 2 h and potential complications after CVS were recorded: presence of apnea, arrhythmias or need for medications. Follow-up period in the Cardiology Clinic was 24 h, including clinical exam of HR, BP, 12-lead ECG (24 h) and blood samples to analyze CK-MB and hsTnI after CVS (8–12 h).

### 2.6. Study Endpoints

The primary endpoint estimated CVS efficacy as the sum of energies delivered by all shocks during one intervention, denoted as cumulative delivered energy (CDE). It is proportional to the total dissipated heat in the heart, generally assumed as the major factor for myocardial injury. The real delivered energy during shock was measured by Defimpulse recorder, considering that delivered energies might differ within ±15% from the selected setting [18].

The secondary CVS efficacy endpoints were:

- Number of delivered shocks;
- Cumulative success rate: presence of sinus rhythm after first shock (1 min), second shock (1 min), and CVS success rate (1 min, 2 h, 24 h) after the last shock;
- Delivered energy of the effective shock.

The secondary CVS safety endpoints were:

- Changes of myocardial-specific biomarkers (CK-MB, hsTnI) at 8–12 h after CVS compared to baseline;
- Proportion of patients with elevated biomarkers;
- Complications after CVS: presence of apnea, arrhythmias, heart-blocks and the need for medication during CVS and 2 h follow-up;
- ST-segment changes (read by cardiologist in lead II, 80 ms after J-point) at 10 s post-shock and 2–5 min post-CVS compared to baseline (10 s pre-CVS);
- Vital signs (HR, BP) after CVS (1 min, 1 h, 2 h).

### 2.7. Statistical Analysis

The statistical analysis was designed for a non-inferiority comparison between the two defibrillation waveforms in respect of their cumulative delivered energies. A non-inferiority margin of 50 J was chosen as the absolute difference in cumulative delivered energy at the end of the CVS study. We estimated that a total of 62 patients (31 per-group) were necessary to show non-inferiority considering a power of 80%, with a one-sided type I error rate of 0.05. Continuous variables were expressed as mean ± standard deviation (std) or median (inter-quartile range) and compared with Student’s t-test or equivalent non-parametric test, respectively. Categorical variables were expressed as percentages and compared using the Chi-square or Fisher’s exact test. The primary and secondary endpoints were reported as mean difference between the treatment groups with 95% confidence intervals (95% CI). Two-tailed *p*-value < 0.05 was considered significant. Statistical analysis was performed with Statistica 7.0 (StatSoft Inc., Tulsa, OK, USA).

## 3. Results

### 3.1. Patients

**Figure 2:**
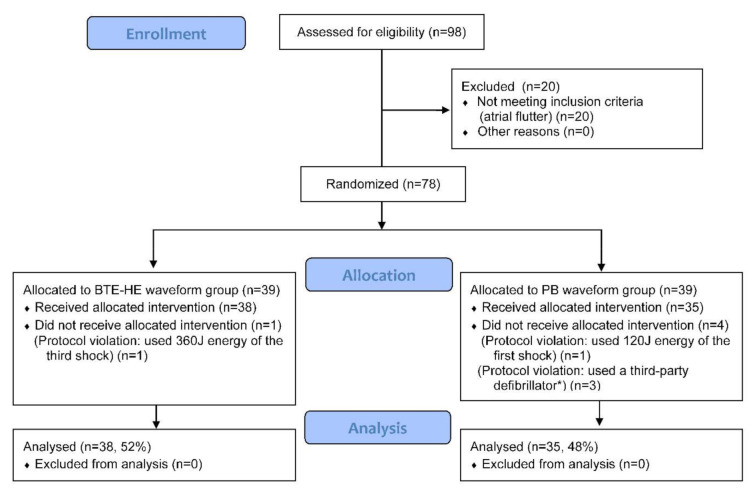
CONSORT flowchart of patient enrollment, allocation and analysis. * The third-party defibrillator was DEFIGARD 4000 (Schiller Médical, Wissembourg, France), embedding the pulsed biphasic technology.

Patients were enrolled from 1 February 2019 to 31 March 2020. In total, 98 patients admitted for elective CVS were screened for participation (Figure 2). Of the 78 randomized patients, an equal number of 39 patients were allocated to both study arms. Five patients did not receive the allocation intervention due to protocol violation (1 BTE-HE, 4 PB) and were excluded following the per-protocol analysis. There were no missing data on the primary endpoint, and no patients were excluded from analysis, involving 38 patients (52%) in the BTE-HE and 35 patients (48%) in the PB group.

### 3.2. Baseline Data

The baseline characteristics were well-balanced between groups (Table 1). No significant differences were found in respect to patients’ demographics, comorbidities and cardiac medications. All patients were in a stable status with normal lab tests, BP and HR before CVS. Deep sedation/anesthesia was achieved by Propofol in similar doses with a median of 90 mg and interquartile range of 80–140 mg (BTE-HE) vs. 80–110 mg (PB), *p* = 0.22.

Each defibrillator used its reference defi-pads. Nevertheless, this led to a non-significant difference of the transthoracic impendence measured before CVS (mean ± std): 87.0 ± 17.3 Ω (BTE-HE) vs. 95.4 ± 16.7 Ω (PB), *p* = 0.057.

### 3.3. Efficacy Endpoints

The primary and secondary efficacy endpoints are presented in Table 2.

**Figure 3:**
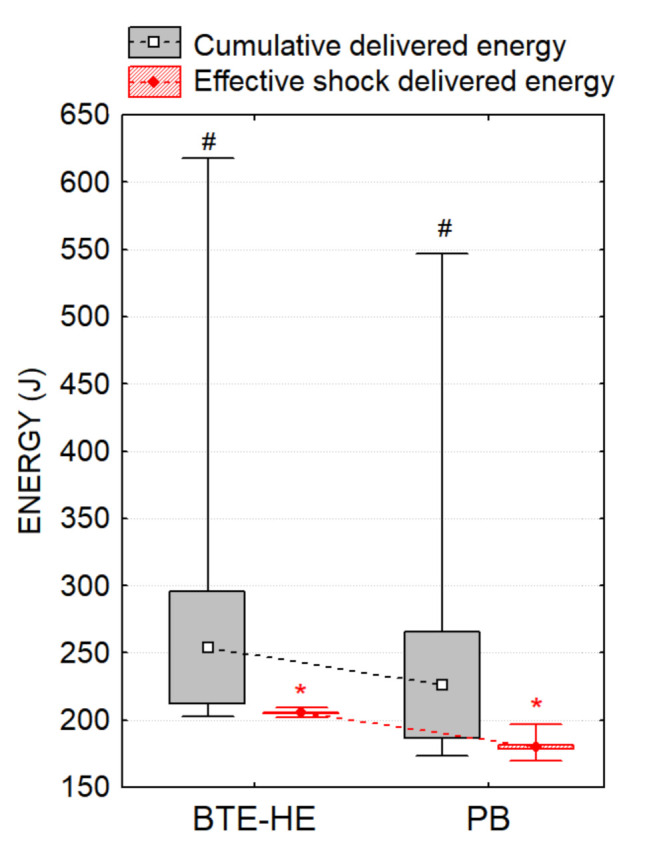
Comparison of BTE-HE and PB waveforms in respect to the delivered energies during CVS: cumulative delivered energy (primary endpoint), delivered energy of the effective shock (secondary endpoint). Plots indicate mean ± 95% CI (box) and min–max range (whiskers). Markers: # *p* = 0.31—Non-inferiority result was proven for the cumulative delivered energies; * *p* < 0.001—Significantly lower delivered energy of the effective shock was found for the PB waveform.

The non-inferiority hypothesis of the primary endpoint was verified (Figure 3), showing that the cumulative delivered energies did not substantially differ among studied waveforms (*p* = 0.31): BTE-HE (mean ± std 253.9 ± 120.2 J, 95%CI 214–293 J, range 203–618 J) vs. PB (mean ± std 226.0 ± 109.8 J, 95%CI 188–264 J, range 173–546 J). A lower cumulative delivered energy of −27.9 J in favor of PB waveform was observed.

Significant difference was found in respect to one secondary efficacy endpoint, indicating lower delivered energy of the effective PB shock by a mean difference of −25.6 J (95% CI −27.1 to −24 J, *p* < 0.001), comparing BTE-HE (mean ± std 205.5 ± 1.53 J, 95% CI 205–206 J, range 202–209 J) with PB (mean ± std 179.9 ± 4.51 J, 95% CI 178–182 J, range 170–197 J), (Figure 3). 

All other tests in Table 2 show similar efficacy outcomes (BTE-HE vs. PB), average number of applied shocks (1.26 ± 0.60 vs. 1.26 ± 0.61, *p* = 0.97), cumulative CVS success rates at 1 min after first shock (84.2% vs. 82.9%, *p* = 0.88), second shock (92.1% vs. 91.4%, *p* = 0.92) and last shock (97.4% vs. 94.3%, *p* = 0.51) efficacies. One patient from the BTE-HE group had an early AF recurrence, leading to a 2 h CVS success rate of 94.7% vs. 94.3%, *p* = 0.93. Within the 24 h follow-up, one patient from the BTE-HE group with severe mitral regurgitation and LV systolic dysfunction had died 8 h after uncomplicated CVS without any preceding complaints or arrhythmias—unwitnessed cardiac arrest with primary recorded rhythm asystole and unsuccessful cardiopulmonary resuscitation by cardiologist and anesthesiologist, leading to a 24 h CVS success rate of 92.1% vs. 94.3%, *p* = 0.72.

### 3.4. Safety Endpoints

The secondary safety endpoints are presented in Table 3. 

Within 2 h follow-up, the overall safety of CVS in both groups was good—apnea, supraventricular tachycardia or ventricular tachycardia/fibrillation were not registered. Transient conduction disturbances (SA and AV blocks) were found in 9 BTE-HE patients (23.7%) and 10 PB patients (28.6%), *p* = 0.64. Only one patient in the PB group was treated with Atropine after CVS, and a temporary pacemaker was not used. Extrasystoles were the most common arrhythmia in the PB group (45.7%, 16/35 patients) vs. BTE-HE (18.4%, 7/38 patients), *p* = 0.012. Indeed, short runs of AF were the most common arrhythmia in the BTE-HE group (15.8%, 6/38 patients) vs. PB (2.86%, 1/35 patients), *p* = 0.062. Additional antiarrhythmic therapy (Amiodarone or Propafenone according to pre-CVS treatment) was applied in 5 patients (BTE-HE) and 4 patients (PB), *p* = 0.41. Within 2 h follow-up, all patients had stable vital parameters within a normal range: mean HR (63–66 min^−1^), systolic BP (116–126 mmHg), diastolic BP (71–74 mmHg), *p* > 0.05. No cases with severe hypotonia (mean BP < 65 mmHg) were recorded. 

Transient post-shock ST-segment changes were rarely recorded (Table 3). After the first shock, they were present in 2 BTE-HE patients (5.56%) and 1 PB patient (2.86%), *p* = 0.57. ST-amplitude shift was not significantly different in both groups (*p* = 0.36). None of the 13 patients treated with a second shock (7 BTE-HE, 6 PB) had post-shock ST-segment changes. Among the 6 patients treated with a third shock (3 BTE-HE, 3 PB), only one in the BTE-HE group had ST-segment changes, with an insignificant amplitude shift (*p* = 0.37). Post-shock ST-segment changes were transient and not registered at 2–5 min or 24 h after CSV. In two PB patients (5.71%), negative T waves were recorded, *p* = 0.14.

Cardiac biomarkers of myocardial injury were available for 69 patients (35 BTE-HE, 34 PB). Their levels before and after CVS did not change significantly, showing post-CVS elevation of hsTnI and CK above normal upper limits in only 2 patients (1 BTE-HE, 1 PB), and no patients with MB elevation.

## 4. Discussion

This was the first prospective randomized clinical trial designed to test the non-inferiority of the cumulative delivered energies of PB and BTE-HE defibrillation waveforms in a fixed energy protocol (200–200–200 J), hypothesizing a difference of 50 J for effective CVS of AF. 

Considering that such energy protocols might accumulate excessive energies, thus presenting a certain risk of myocardial injuries, it was important to assess the safety of both treatments. We found a mean difference of 27.9 J in favor of less energy accumulated by PB waveform. Non-inferiority of PB is validated but superiority over BTE-HE could not be demonstrated (*p* = 0.31). Indeed, effective PB shocks were found to deliver significantly lower energies by mean of 25.6 J (*p* < 0.001) due to design differences in both LP15 and DGT7 defibrillators, which tolerate an energy span of 200 J ± 15% [18]. 

Elective CVS of AF with fixed energy protocol (200-200-200 J) was similarly effective in both arms (*p* > 0.05), reaching >94% success rate for restoration of sinus rhythm at least 24 h after CVS with PB waveform. Considering literature survey findings that CVS dose–response curves concentrate within an efficacy range of 85–95% and mean cumulative energy range of 300–500 J [32], this study neared the upper limit of efficacy range while lowering the threshold of mean cumulative energies to 226 J (PB) and 254 J (BTE-HE). In particular, when compared to two other CVS studies using PB waveforms, the reported success rate of 94.2% (24 h) was similar to 95.4% (1 min) [13] and higher than 67% (4 h) [12], the latter performed with a defective device, and the results of which should be interpreted with caution. A major difference between studies was the used energy protocol: low-escalating [12,13] and fixed (this study). Additionally, in a comparative study of BTE-HE waveforms with low-escalating and maximum-fixed (360 J) energy protocols, Schmidt et al. [14] led to the conclusion of the higher efficacy of fixed-energy protocol. Nevertheless, CVS global efficacy of 88% [14] raises questions for the relevance of accumulating high-energy shocks at 360 J.

Both PB and BTE-HE waveforms showed similar effectiveness with a mean of 1.26 shocks (median of 1 shock) at the fixed energy setting (200–200–200 J). In addition, the number of shocks was lower than other low-escalating studies, reporting a mean of 1.63 PB shocks [13], a mean of 1.9 BTE-HE shocks [9] and a median of 2 BTE-HE shocks [12,14]. Fewer shocks are usually associated with shorter CVS procedure duration, therefore, preferable in respect of reducing the anesthesia dose, the trauma effect on the patient, and the medical staff employment time. Nevertheless, a controversy still exists in AF cardioversion about fixed-energy versus escalating-energy shock protocols.

CVS with up to 3 shocks and up to 600 J was not associated with myocardial injury in our study. No difference in post-shock hsTnI and CK-MB changes were observed regardless of the waveform used (*p* > 0.05). Mild post-CVS elevation was recorded in 1 BTE-HE patient and 1 PB patient. Transient and rare ST-segment changes were provoked in 3 patients (2 BTE-HE, 1 PB) after first shock and 1 patient (BTE) after third shock, disappearing up to 5 min after CVS. There were no cases of other significant deleterious safety events, similar to other CVS studies with fixed energy protocols [14,15].

## 5. Study Limitations

In both arms, patients were excluded from the per-protocol analysis. One patient randomized to the BTE-HE arm received three shocks (200–200–360 J). The reason for protocol violation was the physician’s decision to deliver the last shock with the maximal available energy in LP15 defibrillator on an overweight patient (BMI = 42.1). Nevertheless, the CVS procedure was unsuccessful. Although this case cannot be included in the study, it shows that increasing energy above 200 J for obese patients does not lead to a systematic improvement of care.

Four patients randomized to the PB arm underwent successful CVS with only one PB shock, however were excluded due to physician unawareness of the study protocol: 200 J shock generated with a third-party defibrillator (DG4000, Schiller Médical, Wissembourg, France) in 3 patients; 120 J shock applied to 1 patient. The shock in the latter case was efficient, concluding positively the procedure with PB low-energy shocks.

## 6. Conclusions

The important clinical conclusion of this study is that both PB and BTE-HE waveforms used for cardioversion of AF with fixed energy protocol 200–200–200 J show similarly high efficacy and safety. Although differences in the waveforms’ technical design led to significantly lower delivered energy of the effective PB shocks, no difference in cumulative delivered energy could be highlighted.

## Figures and Tables

**Table 1:** Baseline characteristics of patients in the two study arms. Data were reported as mean ± standard deviation or median (inter-quartile range) for continuous variables and percentages (counts) for categorical variables. All baseline data were not significantly different in both groups (*p* > 0.05).

Baseline Characteristics	BTE-HE Waveform (*n* = 38)	PB Waveform (*n* = 35)	*p*-Value
Age, years	60.9 ± 9.3	64.1 ± 9.5	0.16
Men, % (*n*)	65.8 (25)	60.0 (21)	0.61
Weight, kg	95.2 ± 19.6	93.4 ± 19.3	0.39
Height, cm	175.4 ± 9.34	173.9 ± 9.47	0.50
BMI, kg/m^2^	31.0 ± 5.85	30.9 ± 5.95	0.96
BSA, m²	2.16 ± 0.25	2.14 ± 0.26	0.64
Lean BW, kg	63.0 (52.8–72.2)	61.5 (52.3–71.1)	0.55
Fat BW, kg	28.7 (20.7–45.4)	28.7 (20.5–39.6)	0.94
Chest circumference, cm	114.5 ± 15.2	112.0 ± 13.0	0.46
First-time CVS, % (*n*)	78.9 (30)	77.1 (27)	0.85
Struct heart disease, % (*n*)	86.8 (33)	88.6 (31)	0.82
Heart failure, % (*n*)	42.1 (16)	37.1 (13)	0.66
Diabetes, % (*n*)	21.1 (8)	20.0 (7)	0.91
Hypertension, % (*n*)	55.3 (21)	55.6 (20)	0.98
CAD, % (*n*)	5.13 (2)	5.56 (2)	0.93
Cardiomyopathy, % (*n*)	7.69 (3)	8.33 (3)	0.92
Valvular disease, % (*n*)	17.9 (7)	17.1 (6)	0.93
AF without comorbidities, % (*n*)	12.8 (5)	11.1 (4)	0.82
Persistent AF, % (*n*)	44.7 (17)	42.9 (15)	0.86
First detected AF, % (*n*)	55.3 (21)	57.1 (20)	0.88
COPD, % (*n*)	23.7 (9)	14.3 (5)	0.31
GFR ≤ 60, % (*n*)	23.7 (9)	28.6 (10)	0.64
EGFR, mL/min/1.73 m^2^	70.3 ± 18.3	66.3 ± 15.1	0.30
AF duration, days	42 (30–180)	60 (30–210)	0.58
AF duration < 1 month, % (*n*)	47.4 (18)	34.3 (12)	0.26
AF duration < 1–12 months, % (*n*)	39.5 (15)	51.4 (18)	0.35
AF duration > 12 months, % (*n*)	13.1 (5)	14.3 (5)	0.90
Hb, (norm 120–160), g/L	146.2 ± 12.6	146.7 ± 14.7	0.86
Ht (norm 36–46), % (*n*)	42.5 (39–45)	42 (39–45)	0.54
WBC (norm 4–10), 10^9^/L	7.85 ± 2.24	7.94 ± 1.94	0.86
Glu (norm 4.1–5.9), mmol/L	5.8 (5.4–6.5)	5.7 (5.2–6.9)	0.16
Urea (norm 2.8–7.2), mmol/L	6.6 (5.5–7.7)	6.3 (5.3–7.4)	0.36
Creat (norm 74–110), umol/L	90 (80–111)	94 (85–109)	0.67
Transesophageal Echocardiography			
TEE, % (*n*)	100 (38)	97.1 (34)	0.29
Non-severe echo contrast, % (*n*)	31.6 (12)	31.4 (11)	0.99
LA (anterior-posterior), mm	50.3 ± 9.56	48.5 ± 7.89	0.38
LA < 50 mm, % (*n*)	44.7 (17)	60.0 (21)	0.20
LV TSD, mm	35 (32–42)	34 (30–40)	0.45
LV TDD, mm	52.5 (50–57.5)	52 (48.5–54.5)	0.49
LV TSV, mL	55.5 (41–70)	45 (33–64)	0.28
LV TDV, mL	118 (95–148)	102 (87–130)	0.11
LV EF, % (*n*)	56.5 (49–61)	52 (48–60)	0.99
Normal EF > 50%, % (*n*)	68.4 (26)	71.4 (25)	0.78
ASA Class			
Class 1, % (*n*)	7.89 (3)	5.71 (2)	0.71
Class 2, % (*n*)	39.5 (15)	48.6 (17)	0.44
Class 3, % (*n*)	44.7 (17)	45.7 (16)	0.93
Class 4, % (*n*)	7.89 (3)	0 (0)	0.09
Anesthetic			
Propofol, mg	90 (80–140)	90 (80–110)	0.22
Anticoagulation			
Acenocumarol, % (*n*)	36.8 (14)	20.0 (7)	0.12
Heparin, % (*n*)	5.26 (2)	5.71 (2)	0.93
DOAC, % (*n*)	57.9 (22)	74.3 (26)	0.14
Antiarrhytmic drugs			
Amiodarone, % (*n*)	76.3 (29)	80.0 (28)	0.70
Beta blocker, % (*n*)	73.7 (28)	74.3 (26)	0.95
CCB, % (*n*)	0 (0)	2.86 (1)	0.30
Digitalis, % (*n*)	10.5 (4)	17.1 (7)	0.41
Propafenone, % (*n*)	21.1 (8)	20.0 (7)	0.91
Number AAD (*n*)	1.82 ± 0.56	1.94 ± 0.64	0.37
ACE inhibitor/ARB, % (*n*)	52.6 (20)	54.3 (19)	0.88
Vital signs before CVS			
HR, min^−1^	103.8 ± 19.3	97.3 ± 20.8	0.17
Systolic BP, mmHg	137.0 ± 18.2	139.8 ± 18.1	0.52
Diastolic BP, mmHg	87.6 ± 14.4	88.9 ± 13.3	0.68

AAD: antiarrhythmic drugs, AF: atrial fibrillation, ASA: the American Society of Anesthesiologists Physical Status Classification System, BMI: body mass index, BP: blood pressure, BSA: body surface area, BW: body weight, CAD: coronary artery disease, CCB: calcium channel blocker, COPD: chronic obstructive pulmonary disease, CVS: cardioversion, DOAC: direct oral anticoagulants, EF: ejection fraction, GFR: glomerular filtration rate, EGFR: estimated GFR, HR: heart rate, LA: left atrium, LV: left ventricle, TEE: transesophageal echocardiogram, TDD: telediastolic diameter, TDV: telediastolic volume, TSD: telesystolic diameter, TSV: telesystolic volume.

**Table 2:** Efficacy CVS outcomes. Data are expressed as mean value ± standard deviation for continuous variables, and % (number) for categorical variables. Estimates are reported as mean difference [95% CI].

Efficacy CVS Outcomes	BTE-HE Waveform (*n* = 38)	PB Waveform (*n* = 35)	Mean Difference [95% CI]	*p*-Value
Primary endpoint				
Cumulative delivered energy, J	253.9 ± 120.2	226.0 ± 109.8	−27.9 [−81.8, 26.0]	0.31
Secondary endpoints				
Delivered energy of the effective shock, J	205.5 ± 1.53	179.9 ± 4.51	−25.6 [−27.1, −24.0]	<0.001 *
Number of delivered shocks	1.263 ± 0.601	1.257 ± 0.610	−0.006 [−0.289, 0.277]	0.97
1st shock cumulative success rate at 1 min, % (*n*)	84.2 (32)	82.9 (29)	−1.35 [−18.9, 16.2]	0.88
2nd shock cumulative success rate at 1 min, % (*n*)	92.1 (35)	91.4% (32)	−0.7 [−13.7, 12.3]	0.92
CVS success rate at 1 min, % (*n*)	97.4 (37)	94.3 (33)	−3.1 [−12.5, 6.3]	0.51
CVS success rate at 2 h, % (*n*)	94.7 (36)	94.3 (33)	−0.45 [−11.2, 10.3]	0.93
CVS success rate at 24 h, % (*n*)	92.1 (35)	94.3 (33)	2.2 [−9.8, 14.1]	0.72

* Marked test is statistically significant at *p* < 0.05.

**Table 3:** Safety CVS outcomes. Data are expressed as mean ± standard deviation or median (inter-quartile range) for continuous variables, and % (number) for categorical variables.

Safety CVS Outcomes	BTE-HE Waveform	PB Waveform	*p*-Value
Complications after CVS (2 h follow-up)	(*n* = 38)	(*n* = 35)	
Apnea, % (*n*)	0 (0/38)	0 (0/35)	NA
Rhythm disorders, % (*n*)	34.2 (13/38)	48.6 (17/35)	0.22
Rhythm disorders requiring medication, % (*n*)	38.5 (5/13)	24.0 (4/17)	0.41
E xtrasystoles , % (*n*)	18.4 (7/38)	45.7 (16/35)	0.012 *
AF runs (transient), % (*n*)	15.8 (6/38)	2.86 (1/35)	0.062
Supraventricular tachycardia, % (*n*)	0 (0/38)	0 (0/35)	NA
Ventricular tachycardia/fibrillation, % (*n*)	0 (0/38)	0 (0/35)	NA
Blocks, % (*n*)	23.7 (9/38)	28.6 (10/35)	0.64
SA blocks, % (*n*)	18.4 (7/38)	17.1 (6/35)	0.89
AV blocks, % (*n*)	5.26 (2/38)	11.4 (4/35)	0.34
Blocks requiring Atropine, % (*n*)	0 (0/9)	10 (1/10)	0.34
Blocks requiring pacemaker, % (*n*)	0 (0/9)	0 (0/10)	NA
Post-shock ST-changes	(*n* = 38)	(*n* = 35)	
ST-shift (10 s after shock 1), mm	0.069 ± 0.341	0.014 ± 0.085	0.36
ST-shift (10 s after shock 1), % (*n*)	5.56 (2/38)	2.86 (1/35)	0.57
ST-shift (10 s after shock 2), mm	0 ± 0.00	0 ± 0.00	NA
ST-shift (10 s after shock 2), % (*n*)	0 (0/7)	0 (0/6)	NA
ST-shift (10 s after shock 3), mm	0.333 ± 0.577	0 ± 0.00	0.37
ST-shift (10 s after shock 3), % (*n*)	33.3 (1/3)	0 (0/3)	0.34
ST-shift (2–5 min after CVS), % (*n*)	0 (0/38)	0 (0/35)	NA
T-inverse, (2–5 min after CVS), % (*n*)	0 (0/38)	5.71 (2/35)	0.14
Vital parameters after CVS (2 h follow-up)	(*n* = 38)	(*n* = 35)	0.63
HR (1 min), min^−1^	65.7 ± 12.4	64.3 ± 11.7	0.73
HR (1 h), min ^−1^	65.4 ± 11.5	63.1 ± 10.4	0.45
HR (2 h), min ^−1^	66.1 ± 9.21	64.5 ± 11.8	0.52
Systolic BP (1 min), mmHg	116 ± 15.2	121 ± 17.9	0.25
Systolic BP (1 h), mmHg	117 ± 15.7	120 ± 15.7	0.56
Systolic BP (2 h), mmHg	121 ± 16.9	126 ± 14.4	0.56
Diastolic BP (1 min), mmHg	72.1 ± 9.7	70.6 ± 11.9	0.59
Diastolic BP (1 h), mmHg	71.8 ± 10.7	70.8 ± 10.1	0.74
Diastolic BP (2 h), mmHg	73.8 ± 11.6	73.6 ± 10.5	0.95
Cardiac biomarkers	(*n* = 35)	(*n* = 34)	
hsTnI before CVS (99th percentile = 0.02), ng/mL	0.0044 (0.0027–0.0123)	0.0045 (0.0036–0.0071)	0.36
hsTnI after CVS (99th percentile = 0.02), ng/mL	0.0055 (0.0037–0.0106)	0.0064 (0.0043–0.0133)	0.35
hsTnI change, ng/mL	0.0005 (−0.0009–0.0019)	0.0008 (−0.0004–0.0017)	0.36
Elevated hsTnI after CSV ^a^ , % (*n*)	2.86 (1/35)	2.94% (1/34)	0.98
CK before CVS (ULN = 171), IU/L	75 (50–114)	92 (74–174)	0.008 *
CK after CVS (ULN = 171), IU/L	70 (52–101)	82 (64–115)	0.92
CK change, IU/L	−5 (−15–11)	−17 (−53–10)	0.08
Elevated CK after CSV ^b^ , % (*n*)	2.86 (1/35)	2.94 (1/34)	0.98
MB before CVS (ULN = 25), IU/L	11.0 (8.3–15.0)	11.6 (9.1–15.0)	0.17
MB after CVS (ULN = 25), IU/L	10.7 (9.1–13.7)	10.8 (8.9–12.6)	0.84
MB change, IU/L	0.25 (−2.1–3.85)	−0.2 (−2.1–1.8)	0.24
Elevated MB after CSV ^c^ , % (*n*)	0 (0/35)	0 (0/35)	NA

NA: Not applicable, ULN: upper limit of normal. * Marked test is statistically significant at *p* < 0.05. ^a^ Elevated hsTnI > 0.02 ng/mL (99th percentile of ULN); ^b^ Elevated CK > 171 IU/L(ULN); ^c^ Elevated MB >25 UI/L(ULN) if CK is in normal range or MB > 10% of CK if CK > ULN.

## Data Availability

The data presented in this study are available on request from the corresponding author. The data are not publicly available due to privacy and ethical restrictions.
